# Bow-Legs

**Published:** 1891-07

**Authors:** 


### BOW-LEGS.

Mothers, in training their little ones to walk, seem never to think of how bones grow ; that the bones in a child's legs are soft, half-cartilaginous, and that it is an easy thing to bend them. Hence the need of being careful about having their children walk too soon, or of keeping them on their feet too long when they are first learning to walk. The senseless conduct of many parents in urging their children to walk prematurely is productive of lasting injury. Long before soft bones ought to have any strain put upon them, you will see these poor infants made to stand, and even to walk, and by the time they are fourteen or sixteen months old their little legs have been bent very considerably. Pitiful and permanent deformities produced in this way are seen on every hand. Under a year let the child creep, but do not let it walk, seldom, indeed, stand, and then only for a moment ; and from a year to eighteen or twenty months do not encourage it to walk much, still less set it up on its feet to make it walk.

